# The ProMotion LMU dataset (2022 edition), prostate intra-fraction motion recorded by transperineal ultrasound

**DOI:** 10.1038/s41597-022-01583-0

**Published:** 2022-07-30

**Authors:** Hendrik Ballhausen, Elena Kortmann, Claus Belka, Minglun Li

**Affiliations:** Department of Radiation Oncology, University Hospital, Ludwig-Maximilians-Universität München, Munich, Germany

**Keywords:** Prostate cancer, Prostate, Three-dimensional imaging, Ultrasonography

## Abstract

Infra-fraction motion of the prostate was recorded during 2.385 fractions of image guided radiotherapy (IGRT) in 126 patients, 14 of which were treated by intensity modulated radiation therapy (IMRT), and 112 of which were treated by volumetric arc therapy (VMAT). The prostate was imaged by three-dimensional and time-resolved transperineal ultrasound (4D-US) of type Clarity by Elekta, Stockholm, Sweden. The prostate volume was registered and the prostate position (center of volume) was recorded at a frequency of 2.0 samples per second. This raw data set contains a total of 1.138.024 prostate and patient couch positions over a time span of 158 hours, 25 minutes and 50 seconds of life radiotherapy as exported by the instrument software. This data set has been used for the validation of models of prostate intra-fraction motion and for the estimation of the dosimetric impact of actual intra-fraction motion on treatment quality and side effects. We hope that this data set may be reused by other groups for similar purposes.

## Abstract (summary)

**Table:** 

Measurement(s)	intrafraction motion of the prostate
Technology Type(s)	transperineal ultrasound (4D)
Sample Characteristic - Organism	Homo sapiens
Sample Characteristic - Environment	hospital
Sample Characteristic - Location	Germany

## Background & Summary

Image guided radiotherapy (IGRT) employs various imaging modalities such as computed tomography (CT), cone beam CT (CBCT), stereoscopic x-ray imaging, or ultrasound to locate the target volume and surrounding organs at risk. In particular, the location of the tumour can be used to correct for positioning errors between fractions (inter-fraction) or even in real-time during treatment (intra-fraction).

In our study, we used time-resolved 3D ultrasound (4D-US) to monitor intra-fraction motion of the prostate during primary radiotherapy of adenocarcinoma. We used a trans-perineal robotic probe (Elekta Clarity) which remained fixed to the patient couch and automatically scanned and recorded the prostate position during treatment fractions.

The data we acquired was used to validate the random walk model of intra-fraction motion^1^, investigate a potential impact of patient couch shifts on intra-fraction motion^2^, to estimate the impact of ultrasound probe pressure on intra-fraction drift of the prostate^3^, to show that a shorter treatment time reduces the severity of intra-fraction motion^4^ and to estimate the dosimetric impact of intra-fraction motion on boosts on intra-prostatic lesions^5^.

The dataset described here is a direct extension of the data of the first 28 patients made available in 2019^6^. We hope that it can be reused by other groups for similar purposes.

## Methods

The study is based on 126 patients with adenocarcinoma of the prostate who received a definitive external beam radiotherapy between June 2014 and September 2021 at our department. The first 14 patients of these were treated with IMRT (before May 2015). The latter 112 patients, starting afterwards, received VMAT. The set up remained otherwise unchanged.

The 14 patients in the IMRT group received 35 to 38 fractions of 2.0 Gy each, or 28 fractions of 2.2 Gy plus 10 fractions of 2.0 Gy each. Of the 519 fractions delivered, 358 (69%) were recorded with 4DUS. The decision to record a full fraction (in addition to mandatory daily initial patient setup control by both kV-CBCT and 3DUS) with 4DUS was made by technical personnel based on daily clinical workload. Similarly, the 112 patients in the VMAT group received either 33 to 38 fractions of 2.0 Gy each, 28 fractions of 2.5 Gy each, or 20 fractions of 3.0 Gy each. Of the 2977 fractions delivered, 2027 (68%) were recorded with 4DUS.

Patients were positioned pre-fraction on a 3-DOF robotic couch, matching daily kV-CBCTs to the planning CT. Shifts in vertical, longitudinal, and lateral direction were then corrected by automatic repositioning of the couch. Rotational shifts were not corrected. The new position of the prostate was then used as the reference position. This procedure was repeated before each fraction.

Intra-fraction motion of the prostate was then monitored and recorded by robotic trans-perineal 4DUS using the Clarity system by Elekta, Stockholm, Sweden with an auto-scan probe^7,8^. Patients were placed in supine treatment position, knees on elevated cushions, legs moderately spread. There were no catheters, rectal balloons, spacers or other devices in use to affect intra-fraction motion. The ultrasound probe was fixed to the treatment table and made gel-mediated contact with the perineum at intermediate pressure^3^.

In the IMRT group, an average of 25.5 fractions were recorded per patient (median 27, range 12 to 34). In the VMAT group, an average of 18.0 fractions were recorded per patient (median 17, range 2 to 36). A 15th IMRT patient was excluded from this analysis because he did not complete the course of treatment and had only a single fraction recorded. No other recorded fractions, however, were discarded and there is no indication that the recorded fractions were not representative of an average fraction. The decision to switch the treatment regime from IMRT to VMAT in 2015 was made purely for clinical reasons and blind to the results of this study. While this is a retrospective study, the decision was made to evaluate as soon as an equal number of patients had been recorded with VMAT as with IMRT.

A total 158 hours, 25 minutes and 50 seconds of intra-fraction motion was recorded.

### Human subjects

The study did not involve any experiments on human subjects. All data was generated retrospectively from quality control data acquired in a non-invasive and dose-free fashion during standard treatment independent of this study. Bavarian legislation expressively permits the use of such data for scientific research, cf. Art. 27 (4) of Bayerisches Krankenhausgesetz (BayKrG). All patients included in this study gave written informed consent that their quality control data would be reused for research purposes and would be made available to third parties. Furthermore, the data is completely anonymized and does not contain any identifiable features.

## Data Records

The complete raw data is stored in a public open access repository^9^ at 10.5282/ubm/data.265.

The data is stored in two archive files in.zip and.tar format, respectively, with identical content. The content is organized in 2.385 separate files in comma separated values (CSV) format. The files are named ‘patient_[ppp]_fraction_[nnn].csv’ where [ppp] counts the patients, starting with ‘001’, and [nnn] counts the fractions of each patient, starting over with ‘001’ for each patient.

Table 1 gives an overview of the available data. For example, the data corresponding to the 24 fractions recorded for the first patient is contained in ‘patient_001_fraction_001.csv’ through ‘patient_001_fraction_024.csv’.

**Table 1:** Summary of input data and corresponding data file names.

Patient	Treatment	Day of first fraction	Number of recorded and delivered fractions	Data files - [nn] runs from 01 to the number of recorded fractions
patient_01	IMRT	2014-06-16	24 of 37	patient_001_fraction_[nnn].csv
patient_02	IMRT	2014-07-14	15 of 36	patient_002_fraction_[nnn].csv
patient_03	IMRT	2014-09-29	27 of 37	patient_003_fraction_[nnn].csv
patient_04	IMRT	2014-09-30	29 of 37	patient_004_fraction_[nnn].csv
patient_05	IMRT	2014-09-30	32 of 38	patient_005_fraction_[nnn].csv
patient_06	IMRT	2014-11-25	31 of 37	patient_006_fraction_[nnn].csv
patient_07	IMRT	2015-01-05	19 of 38	patient_007_fraction_[nnn].csv
patient_08	IMRT	2015-01-12	23 of 36	patient_008_fraction_[nnn].csv
patient_09	IMRT	2015-02-02	16 of 38	patient_009_fraction_[nnn].csv
patient_10	IMRT	2015-03-09	22 of 37	patient_010_fraction_[nnn].csv
patient_11	IMRT	2015-04-20	29 of 38	patient_011_fraction_[nnn].csv
patient_12	IMRT	2015-05-05	35 of 38	patient_012_fraction_[nnn].csv
patient_13	IMRT	2015-05-18	34 of 35	patient_013_fraction_[nnn].csv
patient_14	IMRT	2015-05-21	34 of 37	patient_014_fraction_[nnn].csv
patient_15	VMAT	2015-09-29	31 of 36	patient_015_fraction_[nnn].csv
patient_16	VMAT	2016-05-02	28 of 37	patient_016_fraction_[nnn].csv
patient_17	VMAT	2016-05-12	29 of 37	patient_017_fraction_[nnn].csv
patient_18	VMAT	2016-06-02	26 of 36	patient_018_fraction_[nnn].csv
patient_19	VMAT	2016-06-23	35 of 37	patient_019_fraction_[nnn].csv
patient_20	VMAT	2016-07-04	27 of 38	patient_020_fraction_[nnn].csv
patient_21	VMAT	2016-10-10	34 of 37	patient_021_fraction_[nnn].csv
patient_22	VMAT	2016-12-20	31 of 36	patient_022_fraction_[nnn].csv
patient_23	VMAT	2017-01-02	33 of 38	patient_023_fraction_[nnn].csv
patient_24	VMAT	2017-01-19	30 of 38	patient_024_fraction_[nnn].csv
patient_25	VMAT	2017-01-26	31 of 38	patient_025_fraction_[nnn].csv
patient_26	VMAT	2017-01-31	27 of 38	patient_026_fraction_[nnn].csv
patient_27	VMAT	2017-03-21	16 of 38	patient_027_fraction_[nnn].csv
patient_28	VMAT	2017-03-23	17 of 38	patient_028_fraction_[nnn].csv
…	…	…	…	patient_[mmm]_fraction_[nnn].csv

Each CSV files holds a large number of rows, each corresponding to one recorded data point in time, at a sample frequency of about 2.0 Hz.

Table 2 gives an overview of the columns the data is organized into:

**Table 2:** Data format explained.

Field name	Unit	Explanation and remarks
Iso8601Time	s	time stamp in ISO 8601 format (YYYY-MM-DDThh:mm:ss.sss)
SecondsFromMidnight	s	time stamp in second (ss.sss)
XShift	mm	prostate position (x-axis)
YShift	mm	prostate position (y-axis)
ZShift	mm	prostate position (z-axis)
CouchRelativeX	mm	couch position (x-axis)
CouchRelativeY	mm	couch position (y-axis)
CouchRelativeZ	mm	couch position (z-axis)
Quality	1	data quality indicator
HasCouchRelativePosition	TRUE/FALSE	should always be TRUE

**Iso8601Time** is a time stamp in ISO 8601 format (YYYY-MM-DDThh:mm:ss.sss). Its absolute value is not meaningful, as the workstations internal clock may or may not have been correctly set at all times (in particular, regional daylight saving settings). However, the time stamps are essential in calculating relative durations. It is useful to define the begin of treatment as arbitrary zero.

**SecondsFromMidnight** is a time stamp in seconds (and milliseconds in the decimal places). As before, it is useful to define durations and one should choose an arbitrary zero.

**XShift** denotes the recorded position of the prostate on the **longitudinal** axis in units of mm. As the patient is lying on the treatment couch, this axis is horizontal in the laboratory frame of reference and points away from the gantry. Increasing values describe a motion in caudal direction, away from the gantry. Decreasing values describe a motion in cranial direction, towards the gantry. The absolute value of this quantity is not meaningful, one should define a suitable zero.

**YShift** denotes the recorded position of the prostate on the **lateral** axis in units of mm. As the patient is lying on the treatment couch, this axis is horizontal in the laboratory frame of reference and parallel to the gantry. Increasing values describe a motion towards the left side of the patient. Decreasing values describe a motion in towards the right side of the patient.

**ZShift** denotes the recorded position of the prostate on the **vertical** axis in units of mm. As the patient is lying on the treatment couch, this axis is also vertical in the laboratory frame of reference and points up. Increasing values describe a motion in anterior direction, or upwards. Decreasing values describe a motion in posterior direction, or downwards. The absolute value of this quantity is not meaningful; one should define a suitable zero.

**CouchRelativeX**, **CouchRelativeY**, **CouchRelativeZ** describe the **absolute** position of the patient couch with respect to the laboratory frame of reference, again on the longitudinal, lateral, and vertical axis and with the same orientations as before.

**XShift**, **YShift**, and **ZShift** decribe the position of the prostate **relative** to the ultrasound probe, which is fixed to the patient couch.

Thus, if one is interested in the physiological motion of the prostate, one should simply consider XShift, YShift, and ZShift. However, if one is interested in the absolute motion of the prostate, e.g. relative to the treatment beam, one should consider **XShift + CouchRelativeX**, **Yshift + CouchRelativeY**, and **ZShift + CouchRelativeZ**, respectively.

**Quality** is an indicator representing the quality of the rigid registration calculation, based on machine learning^10^. It ranges from zero (least reliable fit) to one (best possible fit).

**HasCouchRelativePosition** should always read ‘TRUE’.

## Technical Validation

The spatial resolution of the ultrasound system is specified by the manufacturer to about 0.2 mm^8^. The overall geometric inaccuracy of a very similar setup due to inherent technical limitations was measured to be 0.6 mm laterally, 0.7 mm vertically, 0.5 mm longitudinally, and 1.1 mm radially (‘vector length’ or Euclidean ‘3D-distance’; the square root of the sum of squares of the three axes) consisting of random errors (per single measurement point) and systematic errors (effectively, per fraction)^11^. The temporal resolution of the device is specified to about 2 Hz^8^; data was in fact recorded at 1.6 Hz on average.

The particular setup used in this study has been characterised before in detail^12^. The discrepancy between ultrasound localisation and implanted gold markers detected by CBCT was 0.0 ± 1.7 mm laterally, 0.2 ± 2.0 mm longitudinally, and 0.3 ± 1.7 mm vertically. Using implanted gold markers as a reference, systematic errors for ultrasound localisation were 1.2 mm, 1.1 mm, and 0.9 mm; and random errors were 1.4 mm, 1.8 mm, and 1.6 mm, on lateral, longitudinal, and vertical axes, respectively. The majority of these errors stems from inter-modality comparisons; within the modality accuracy and repeatability were generally sub-millimeter. The setup was routinely gauged during weekly QA.

The motion management system of the Clarity system was used to shut off the beam whenever the prostate position exceeded a certain threshold per axis. In such cases, the table position was manually corrected and the prostate position checked before treatment was resumed. However, this motion of the table did not produce any excessive acceleration that could have caused prostate motion of its own. In particular, we checked^2^ that the table motion was not visible in the prostate motion data as the prostate position was recorded relative to the table and not in absolute room coordinates. Therefore, our simulation resembles a situation before or without active prostate motion management and correction.

## Usage Notes

In our own analysis, we first visually inspected the prostate trajectories one by one. The data features end-of-fraction outliers, e.g. caused by patients leaving their position after treatment is stopped. We opted, however, to leave the full original raw data in the deposited dataset, including outliers.

It is useful to resample the data to reduce high frequency noise and to equalize the time intervals (readouts do not occur perfectly equitemporal). In our analysis, we chose bins of five-second intervals.

The data is publicly available from Open Data LMU under CC BY 4.0 license. There are no access controls in place. Use of the data is not limited.

## Data Availability

No custom code was used in the generation or processing of datasets. All data is provided as ASCII text in CSV format and can be processed without custom code. An optional interactive Excel worksheet for convenient browsing, importing and resampling of the data is available upon request from the corresponding author.
